# High level expression of glucocorticoid receptor (GR) is linked to aggressive tumor features, early biochemical recurrence, and genetic instability in prostate cancer

**DOI:** 10.1038/s41391-025-01046-8

**Published:** 2025-11-05

**Authors:** Neele Heckmann, Henning Plage, Ronald Simon, Maximilian Lennartz, Christoph Fraune, Frank Jacobsen, Till Krech, Patrick Lebok, Sarah Minner, Eike Burandt, Till S. Clauditz, Waldemar Wilczak, Guido Sauter, Natalia Gorbokon, Morton Freytag, Florian Lutz, Viktor Reiswich, Florian Viehweger, Viktoria Chirico, Hans Heinzer, Alexander Haese, Thorsten Schlomm, Andreas Marx, Markus Graefen, Stefan Steurer, Christian Bernreuther, Bernhard Ralla, David Dum, Andrea Hinsch, Simon Kind, Andreas M. Luebke, Anne Menz, Katharina Möller, Ria Schlichter, Sören Weidemann, Barbara Erber, Nadine Biernath, Sarah Weinberger

**Affiliations:** 1 Martini-Clinic, Prostate Cancer Center, University Medical Center Hamburg-Eppendorf, Hamburg, Germany; 2 Institute of Pathology, University Medical Center Hamburg-Eppendorf, Hamburg, Germany; 3 Department of Urology, Charité - Universitaetsmedizin Berlin, Berlin, Germany; 4 Department of Pathology, Academic Hospital Fuerth, Fuerth, Germany

**Keywords:** Diseases, Prognostic markers

## Abstract

**Background:**

The glucocorticoid receptor (GR) is a nuclear receptor protein for cortisol and other glucocorticoids and regulates the transcription of thousands of genes involved in metabolism, development, stress and inflammatory response. In prostate cancer, GR may confer resistance to anti-androgen receptor therapies by bypassing AR blockade. However, only few data are available on the prognostic role of GR expression in prostate cancer.

**Methods:**

To estimate the prognostic value of GR, a tissue microarray containing 17,747 prostate cancers with associated follow-up and molecular data was analyzed by immunohistochemistry.

**Results:**

All patients had undergone radical prostatectomy. GR immunostaining was found in 10,832 (89.1%) of 12,125 interpretable tumors, including 48.5% with weak, 29.8% with moderate and 11% with strong staining intensity. Increased GR staining was strongly linked to adverse feature of the disease, including high tumor stage (pT), high classical and quantitative Gleason grade, presence of nodal metastases (pN+), a positive surgical margin (R1) status, and early biochemical recurrence (*p* < 0.0001 each). A multivariate analysis showed that the prognostic value of strong GR staining was independent of pT, Gleason grade, pN and R status. High level GR staining was significantly linked to TMPRSS2:ERG fusion (*p* < 0.0001) and high androgen receptor expression (*p* < 0.0001 each). A combined analysis of the impact of GR and AR on patient prognosis identified the best prognosis for AR^neg^/GR^neg^ cancers while AR^pos^/GR^pos^ cancers showed the worst prognosis (*p* < 0.0001). Moreover, high GR expression was a strong predictor of poor prognosis in AR low, AR intermediate and AR high cancers (*p* < 0.0001 each).

**Conclusion:**

High level expression of GR is strongly linked to prostate cancer aggressiveness in uni- and multivariate analysis. GR immunohistochemistry – alone or in combination with other markers – holds great potential to identify patients with a high risk for tumor progression.

## Introduction

Prostate cancer is the most prevalent cancer in men in Western societies. Although the majority of prostate cancers behave in an indolent manner, a small subset is highly aggressive and requires extensive treatment. Established preoperative prognostic parameters are limited to Gleason grade and tumor extent on biopsies, serum PSA (prostate-specific antigen) levels, and clinical stage. These parameters are statistically strong, but not sufficient to enable optimal treatment decisions in every patient. It is, thus, hoped that a better understanding of disease biology will eventually lead to the identification of clinically applicable molecular markers that enable a more reliable prediction of prostate cancer aggressiveness.

The glucocorticoid receptor (GR) is a member of the nuclear receptor (NR) superfamily subgroup 3 which also contains the other steroid receptors androgen receptor (AR), mineralocorticoid receptor (MR), progesterone receptor (PR), and the two estrogen receptors (ERα and ERβ). GR is the most relevant receptor protein for cortisol and other glucocorticoids [1]. Depending on ligand binding and recruitment of context specific transcriptional coregulators, GR modulates either activation or repression of the transcription of a broad range of different genes involved in development, metabolism, stress and inflammatory responses [2]. GR expression plays a complex role in tumor biology which goes beyond its effects on the immune system [3, 4]. In several tumor types, both an oncogenic and a tumor suppressive function of GR has been found depending on specific tumor conditions [4, 5]. The role of GR in prostate cancer biology appears to be complex. While GR activation inhibited tumor angiogenesis [6] and reduced proliferation in primary hormone-sensitive prostate cancer cell lines [7], there is also evidence for an oncogenic role of GR. GR for example promoted prostate cancer cell survival by upregulation of anti-apoptotic proteins such as SGK-1 and downregulation of autophagy [8, 9], undermining the efficacy of cancer treatment.

Most of the current clinical interest in GR expression in cancer comes from its potential as a druggable target. This is especially true for castration resistant prostate cancer (CRPC) where GR overexpression is a mechanism for androgen resistance [8]. Concordant to these findings, preclinical studies provided evidence that GR pathway inhibition leads to a resensitization to antiandrogen therapy and also docetaxel [10]. Several GR inhibitors, such as Mifepristone, Exicorilant and ORIC-101, have already been tested in CRPC patients in combination with enzalutamide. However, none of these has so far shown a significant clinical benefit [11]. Low GR expression levels in significant subsets of the treated tumors were considered a potential explanation for this poor response to GR blockade [11].

To better understand the potential clinical impact of GR protein expression in prostate cancer we took advantage of our large prognosis tissue microarray (TMA) with its attached database on clinical, pathological and molecular data and studied patterns of GR expression in more than 12,000 prostate cancer patients by immunohistochemistry (IHC).

## Material and methods

### Patients

Radical prostatectomy specimens were from 17,747 patients, undergoing surgery between 1992 and 2015 at the Department of Urology and the Martini Clinic at the University Medical Center Hamburg-Eppendorf. Follow-up data were obtained for 14,464 of these patients with a median follow-up time of 48 months (range: 1–275 months; Supplementary Table 1). Prostate specific antigen (PSA) values were measured following surgery and PSA recurrence was defined as the time point when postoperative PSA was at least 0.2 ng/ml and increasing at subsequent measurements. Histopathological data were retrieved from the patient files, including tumor stage, Gleason grade, nodal stage and resection margin status. In addition to the classical Gleason categories, quantitative Gleason grading was performed as described before [12]. In brief, for every prostatectomy specimen, the percentages of Gleason 4 patterns in cancerous tissues were estimated during the regular process of pathologic interpretation. Gleason 3 + 4 and 4 + 3 cancers were subdivided according to their percentage of Gleason 4 in 8 subgroups: 3 + 4 ≤ 5% Gleason 4, 3 + 4 6–10%, 3 + 4 11–20%, 3 + 4 21–30%, 3 + 4 31–49%, 4 + 3 50–60%, 4 + 3 61–80% and 4 + 3 > 80% Gleason 4. Furthermore, separate groups were defined by the presence of a tertiary Gleason 5 pattern, including 3 + 4 Tert. 5 and 4 + 3 Tert. 5. The TMA manufacturing process was described earlier in detail [13]. In short, one 0.6 mm core was taken from a cancer containing tissue block from each patient. The tissues were distributed among 27 TMA blocks, each containing 144–522 tumor samples. For internal controls, each TMA block also contains various control tissues, including normal prostate tissue. The molecular database attached to this TMA contains results on V-ets avian erythroblastosis virus E26 oncogene homolog (ERG) protein expression in 12,789 and ERG break apart FISH analysis in 7036 tumors. Deletion data were available of 10q23 (PTEN) from 5241 tumors, of 6q15 (MAP3K7) from 4722 tumors, of 5q21 (CHD1) from 6031 tumors, of 3p13 (FOXP1) from 5513 tumors, of 8p21 from 5489 tumors, of 12p13 (CDKN1B) from 4887 tumors, of 12q24 from 5625 tumors, of 13q14 from 5915 tumors, of 16q24 from 4413 tumors, of 17p13 from 6249 tumors, and of 18q21 from 5332 tumors [14]. The use of archived diagnostic left-over tissues for manufacturing of tissue microarrays and their analysis for research purposes as well as patient data analysis has been approved by local laws (HmbKHG, §12,1) and by the local ethics committee (Ethics commission Hamburg, WF-049/09). All work has been carried out in compliance with the Helsinki Declaration.

### Immunohistochemistry

Freshly prepared TMA sections were immunostained on one day in one experiment. Slides were deparaffinized with xylol, rehydrated through a graded alcohol series, and exposed to heat-induced antigen retrieval for 5 min in an autoclave at 121 °C in pH 7.8 buffer. Endogenous peroxidase activity was blocked with Dako Peroxidase Blocking Solution™ (Agilent, CA, USA; #52023) for 10 min. Primary antibody specific against GR (recombinant rabbit monoclonal, HMV304; ardoci GmbH, Hamburg, Germany) was applied at 37 °C for 60 min at a dilution of 1:150. Bound antibody was then visualized using the EnVision Kit™ (Agilent, CA, USA; #K5007) according to the manufacturer’s directions. The sections were counterstained with haemalaun. For tumor tissues, the percentage of GR positive tumor cells was estimated, and the staining intensity was semi-quantitatively recorded (0, 1+, 2+, 3+). For statistical analyses, the staining results were categorized into four groups as follows: Negative: no staining at all, weak staining: staining intensity of 1+ in ≤70% or staining intensity of 2+ in ≤30% of tumor cells, moderate staining: staining intensity of 1+ in >70%, staining intensity of 2+ in >30% but in ≤70% or staining intensity of 3+ in ≤30% of tumor cells, strong staining: staining intensity of 2+ in >70% or staining intensity of 3+ in >30% of tumor cells. For survival analyses including combinations of GR and androgen receptor expression data, GR expression was categorized into negative, low (weak or moderate staining) and high (strong staining).

### Statistics

Statistical calculations were performed with JMP^®^ 17 software (SAS Institute Inc., NC, USA). Contingency tables and the chi²-test were performed to search for associations between molecular parameters and tumor phenotype. Survival curves were calculated according to Kaplan–Meier. The Log-Rank test was applied to detect significant differences between groups. Cox proportional hazards regression analysis was performed to test the statistical independence and significance between pathological, molecular and clinical variables. Separate analyses were performed using different sets of parameters available either before or after prostatectomy.

## Results

### Technical issues

A total of 12,152 (68.5%) tumor samples were interpretable in our TMA analysis. Reasons for non-informative cases included lack of tissue samples or absence of unequivocal cancer tissue in the TMA spot in 5595 (31.5%) tumors.

### GR expression in normal and cancerous prostate tissues

**Fig. 1:**
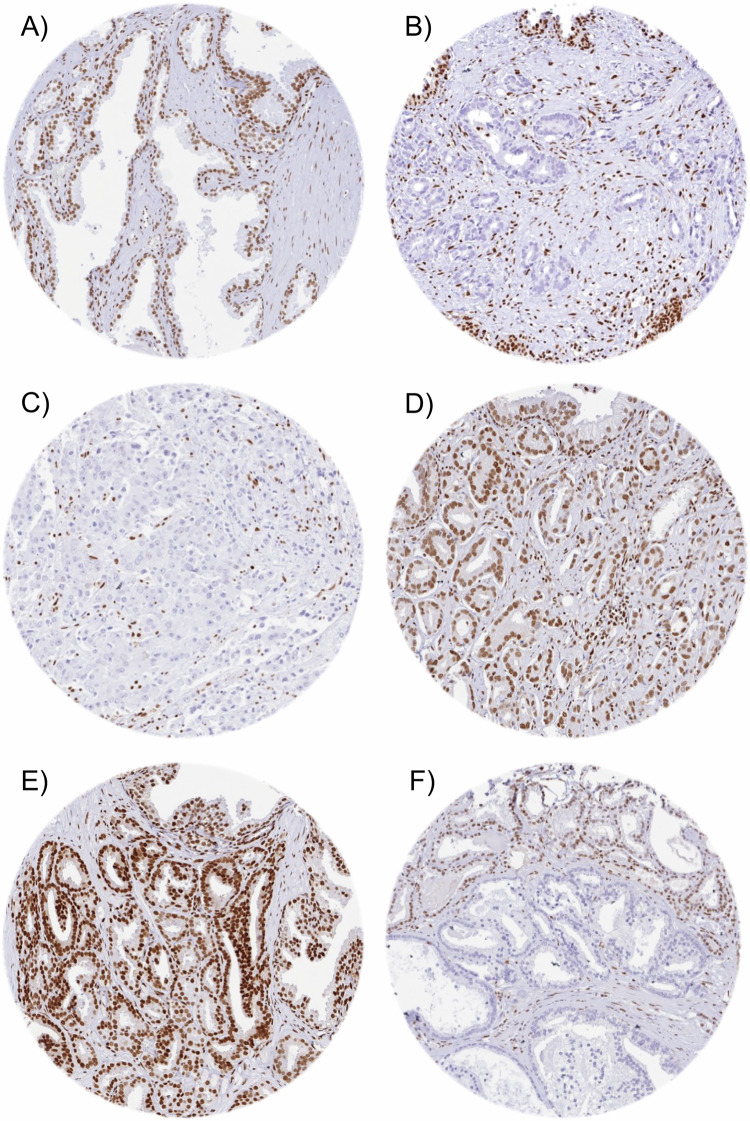
Representative images of GR immunostaining. GR staining in normal (**A**) and cancerous glands with negative (**B** and **C**), moderate to strong (**D** and **E**) and heterogenous (**F**) GR expression.

**Fig. 2:**
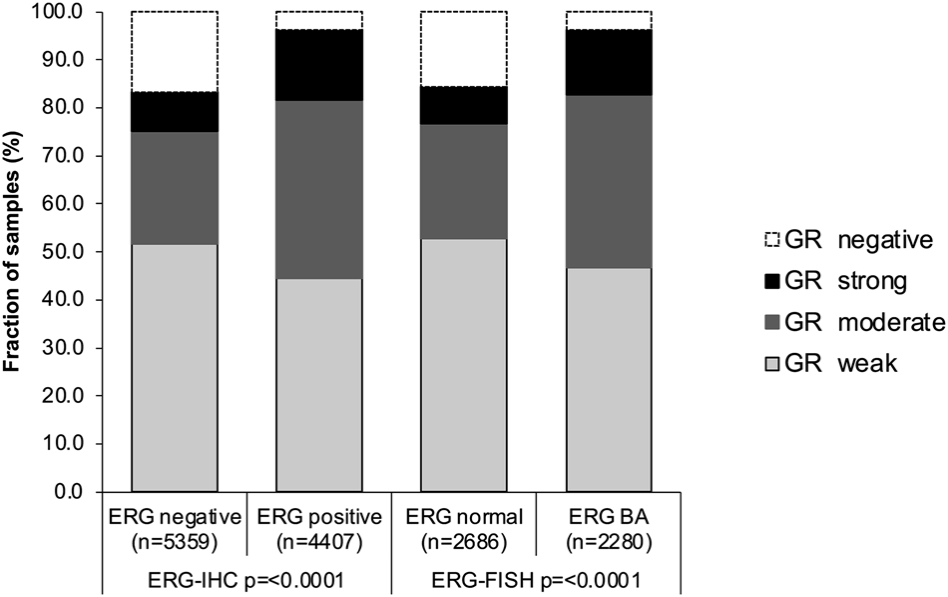
Association between positive GR Immunostaining and ERG-status (IHC/FISH) in all cancers.

Normal prostate glands showed moderate nuclear staining of luminal cells and strong staining of basal and stromal cells. In cancer, nuclear GR positivity was seen in 10,832 (89.1%) of cases including 48.5% with weak, 29.8% with moderate, and 11% with strong staining. Samples with adjacent normal and cancerous glands revealed that both reduced expression and overexpression can occur in cancer cells as compared to normal prostate glands. Representative images of GR immunostainings in normal and cancerous glands are shown in Fig. 1. Among the tumors with interpretable GR staining, data on ERG FISH were available in 4966, and on ERG IHC in 9766 cancers. A higher level of GR staining was linked to *TMPRSS2:ERG* fusion positive prostate cancers. For example, moderate to strong GR staining was seen in 31.5% of ERG IHC negative, but in 51.6% of ERG IHC positive cancers (Fig. 2).

### Tumor phenotype and PSA recurrence

**Fig. 3:**
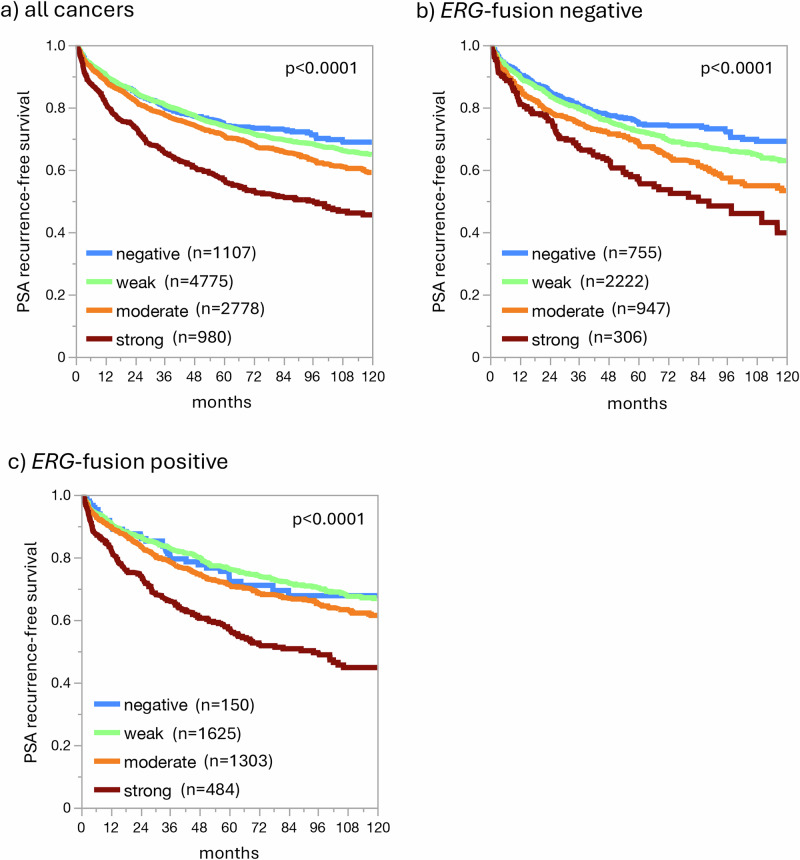
Prognostic impact of GR expression in prostate cancer. Association between GR expression and biochemical recurrence in **a** all cancers, **b** *ERG* fusion negative cancers, **c** *ERG* fusion positive cancers.

Increased GR expression was significantly linked to advanced tumor stage, high classical and quantitative Gleason grade, and positive nodal status (*p* < 0.0001 each; Table 1). Most of these associations also held true in subset analyses of ERG negative or ERG positive cancers (Supplementary Table 2 and 3). Follow-up data were available for 9590 patients with interpretable GR staining. High GR expression was strongly linked to early PSA recurrence in all tumors (Fig. 3a, *p* < 0.0001) as well as in subsets of *ERG*-fusion negative (Fig. 3b, *p* < 0.0001) and positive cancers (Fig. 3c, *p* < 0.0001). The prognostic role of GR expression was retained in the subgroups of Gleason 3 + 4 = 7 (*p* < 0.0001) and Gleason ≥4 + 4 (*p* = 0.0369), but not in the subsets of Gleason 3 + 3 = 6 and 4 + 3 = 7 cancers (Supplementary Fig. 1a–d). However, the analysis of tumor subsets with comparable quantitative Gleason grade did not indicate a significant prognostic impact of GR expression in any group (Supplementary Fig. 2a–j).

**Table 1:** Association between GR immunostaining results and prostate cancer phenotype in all cancers.

			GR immunostaining				
		*n* evaluable	Negative (%)	Weak (%)	Moderate (%)	Strong (%)	*p* value
All cancers		12152	10.9	48.5	29.8	11	
Tumor stage	pT2	7401	12	50.2	29.1	8.7	<0.0001
	pT3a	2890	9.6	47	31.5	11.9	
	pT3b-4	1805	8.5	43.4	30.1	18.1	
Gleason grade	≤3 + 3	2059	13.9	54.6	26.1	5.4	<0.0001
	3 + 4	6416	10.6	49.1	30.7	9.6	
	3 + 4 Tert.5	613	8	49.3	31	11.7	
	4 + 3	1250	9.2	42.4	32.2	16.2	
	4 + 3 Tert.5	937	8.8	42.9	30.6	17.7	
	≥4 + 4	758	13.2	44.5	24.7	17.7	
Quantitative Gleason	3 + 4 ≤5%	1531	12.1	52.4	28	7.5	<0.0001
	3 + 4 6-10%	1586	10.5	52.1	28.8	8.6	
	3 + 4 11-20%	1417	10.7	47.5	32.6	9.2	
	3 + 4 21-30%	731	8.8	47.2	33.7	10.4	
	3 + 4 31-49%	617	10.4	47.2	29.5	13	
	4 + 3 50-60%	490	8	49.3	31	11.7	
	4 + 3 61-80%	441	9.4	41	33.5	16.1	
	4 + 3 >80%	121	9.3	43.3	31.7	15.6	
Lymph node metastasis	N0	7511	10.5	47.7	30.6	11.2	<0.0001
	N+	999	8.7	40.7	31.3	19.2	
Preop. PSA level (ng/ml)	<4	1360	7	48.8	31	13.2	<0.0001
	4–10	7048	10.3	48.9	30.3	10.5	
	11–20	2658	12.4	47.2	29.7	10.7	
	>20	1008	16	48.4	24.8	10.8	
Surgical margin	Negative	9513	10.8	48.7	30.3	10.2	<0.0001
	Positive	2591	11.3	47.4	27.9	13.3	

### Multivariate analysis

Four different multivariate analyses were performed to evaluate the clinical relevance of GR expression in different scenarios (Supplementary Table 4). Scenario 1 evaluated all postoperatively available parameters including pT, pN, surgical margin status, preoperative PSA value and Gleason grade obtained on the prostatectomy specimen. In scenario 2, all postoperatively available parameters except pN were included. The rationale for this approach was that the indication and extent of lymph node dissection is not standardized in the surgical therapy of prostate cancer and may introduce a bias towards high grade cancers. Two additional scenarios were to model the preoperative situation as accurately as possible: Scenario 3 included GR expression, preoperative PSA, clinical tumor stage (cT stage) and Gleason grade obtained on the prostatectomy specimen. Since postoperative determination of a tumor’s Gleason grade is more precise than the preoperatively determined Gleason grade (subjected to sampling errors) this parameter was replaced by the preoperative Gleason grade obtained on the original biopsy in Scenario 4. GR expression provided significant prognostic value beyond the established parameters in all of the described scenarios, particularly in the preclinical scenario 4. This preclinical prognostic value also held true for the subgroups of ERG negative and ERG positive cancers.

### Androgen receptor, chromosomal deletions

Data on both GR and AR were available for 5621 cancers. There was a strong positive association between AR expression and GR expression in all cancers as well as in subsets of ERG negative and ERG positive cancers (*p* < 0.0001 each; Supplementary Fig. 3). A combined analysis of the prognostic role of AR and GR expression revealed that AR^neg^/GR^neg^ tumors and GR^low^ tumors had beneficial outcomes while prognosis significantly worsened for tumors having both (AR^high^/GR^high)^ or only one of the two receptors highly expressed (AR^high^/GR^neg^ or AR^neg^/GR^high^, Supplementary Fig. 4, *p* < 0.0001; Supplementary Fig. 5). Moreover, a high GR expression was always associated with aggressive disease course in cancers with comparable AR expression levels (negative, low and high). A comparison with 11 different common deletions in prostate cancer revealed an association between high GR expression and deletions of PTEN (*p* < 0.0001) as well as the six additional genomic loci 3p13, 8p21, 17p13 (*p* < 0.0001 each), 12p13 (*p* = 0.0014), 16q24 (*p* = 0.0031) and 18q21 (*p* = 0.0213). The majority of these associations could also be found in subset analyses of ERG negative or ERG positive cancers (Supplementary Fig. 6).

## Discussion

The analysis of more than 17,000 prostate cancers revealed that both a complete loss of detectable expression and a significant overexpression of GR as compared to adjacent normal prostate epithelium occurs in significant portions of treatment naïve tumors. This underlines the complex role of GR in prostate cancer development. Our overall GR positivity rate of 89.1% lies within the upper range found in current literature. Previous IHC studies had found GR positivity in 20–100% in cohorts of 11–168 cancers [7, 15–21], and in 100% of 14 prostate cancer metastases [18]. Using the same antibody and experimental conditions, we found a comparable rate of GR positivity (93%) in 129 recurrent prostate cancers, suggesting that GR expression is retained in recurrent and metastatic lesions [22]. It should be noted that GR is one of several proteins whose significance in cancer can only be assessed using in situ methods such as IHC, as GR is ubiquitously expressed in all cell types. When analyzing desintegrated tissues (e.g. for RNA sequencing), there is therefore a high risk that non-neoplastic cells (like stromal cells) in the tissue sample will significantly influence the results. However, varying positivity rates in different IHC studies are likely due to the use of different antibodies, staining protocols, and criteria for defining positivity as well as patient selection criteria. The assay used in our study had previously been validated according to the guidelines of the international working group for antibody validation (IWGAV) by comparing with RNA data and with an independent second antibody in 76 different normal tissue types [22].

The strong and independent association of high GR expression with unfavorable tumor phenotype and poor clinical outcome represents the key finding of our study. In line with our data, Guo et al. also reported a positive correlation between high GR expression and a high tumor stage, unfavorable Gleason score, and shorter PSA progression-free survival [16]. However, Xie et al. [23] did not find a significant association of GR expression with a more aggressive phenotype in subrenal capsule xenografts, and Hata et al. [24] even found an inverse correlation between GR staining intensity and tumor stage in a cohort of 101 prostate cancers. Data from other tumor entities may suggest a tumor type dependent role of GR in cancer progression. A link between high GR expression levels and poor prognosis and/or unfavorable tumor phenotype has been reported for ovarian [25], endometrial [26], gastric [27], and ER-negative breast cancer [28], as well as hepatocellular [29] and salivary duct carcinoma [30]. Significant associations between low GR expression and poor prognosis were described for myeloma [31], lymphoblastic leukemia [32], as well as adrenocortical [33], thymic [34], bladder [35] and small cell lung cancer [36]. Using the same IHC assay as in this study, we had also found a strong correlation between low GR expression and poor prognosis in clear cell renal cell carcinomas [22].

That our combined analysis of the prognostic role of AR and GR revealed a particularly strong prognostic impact of GR expression in tumors with only low AR levels is consistent with a synergistic role of these receptors in prostate cancer. These data are in line with studies describing GR overexpression as a mechanism of androgen resistance by bypassing the AR pathway [8, 18, 37]. Experiments on prostate cancer cell lines identified a strong GR-upregulation under ADT [37], a strong anti-proliferative effect of the GR-inhibitor mifepristone in cells with high GR and low AR expression [38], and a suppressed GR expression in cells with high AR activity by binding of ligand-activated AR to a negative response element near the GR gene locus [10, 23].

Interestingly, low levels of GR appeared to be more beneficial than negative or high GR levels in AR positive cancers (low-high), suggesting potential synergistic effects between the receptors which are lost if one receptor is missing, or both are highly expressed. It is known that crosstalk of specific transcription factors - such as steroid receptors –sustained the tumor-suppressive function of one or both transcription factors in a very finely tuned system and that disruption of these interactions may contribute to increased tumorigenesis. In AR positive prostate cancer cells, GR at normal expression levels act as a tumor suppressor based on a direct crosstalk by pioneer factors, identical DNA-binding sites and mutual transcriptional regulation [7]. Whereas, upregulation of GR in AR positive prostate cancer cells – especially if both receptors are upregulated - can amplify transcriptional output and promote tumor cell survival more effectively than either receptor alone [39]. On the other hand, GR negativity could be used as a marker of high AR activity as AR negatively regulates GR expression [23]. Furthermore, it is assumed that the synergistic effect of AR and GR in prostate cancer cells depends on the molecular background and progression status [40].

It is of note that some of our data are in conflict with these experimental data as we found a strong positive correlation of AR and GR expression in our in vivo analysis of 5621 prostate cancers with data on both receptors. However, others have also found a positive correlation of AR and GR expression in prostate cancer under antiandrogen treatment [18]. In these tumors, GR-mediated elevation of AR-stability was proposed to represent a possible underlying mechanism [41]. Molecular mechanisms, other than castration-resistance, have also been described as potential drivers of tumor progression in GR positive prostate cancer. These include, for example, GR-induced activation of pro-metastatic processes such as cytoskeleton remodeling via CALD1 expression [42], epithelial-mesenchymal transition [43, 44], upregulation of anti-apoptotic proteins like SGK-1 [8], and downregulation of autophagy [9].

Data from previous studies also allowed a comparison of our GR data with some of the most common genomic alterations of prostate cancer. The *TMPRSS2:ERG* fusion effects about 50% of prostate cancers [45], is induced by AR-signaling [46], and regulates a huge variety of genes [47]. However, the promoter of the NR3C1 gene encoding the GR has no ERG binding site, suggesting that the significantly higher GR levels in cancers with a *TMPRSS2:ER*G fusion are caused by secondary effects of the transcriptional reprogramming associated with ERG activation. That the prognostic effect of GR expression was stronger in ERG negative than in ERG positive cancers is in line with a modifying role of the ERG status on the prognostic role of other molecular features. Androgen dependent ERG expression results in an altered expression of more than 1600 genes in affected prostate epithelial cells [48]. In earlier studies using our prostate cancer TMA, several molecular parameters were only prognostic in either ERG positive [49, 50] or ERG negative cancers [51, 52]. That GR expression was associated with six of eleven analyzed chromosomal deletions in ERG negative and with five deletions in ERG positive cancers could potentially be explained by a higher level of oxidative stress in genomically instable prostate cancer cells [53]. Especially long-term GR activation has been associated with stimulation of OXPHOS and accumulation of ROS [54–56]. As some of the GR-associated deletions may represent key drivers for androgen-independent prostate cancer subtypes [57], GR upregulation might just reflect an alternative pathway (other than AR signaling) in some deleted cancers. A current study by Yip et al. [58] has linked PTEN activity to GR expression levels providing a potential explanation for the particularly strong association we found with PTEN deletion. In this study, loss of PTEN protein-phosphatase activity resulted in higher GR levels in breast cancer mouse models, while a loss of lipid-phosphatase activity alone led to reduced GR levels.

Given the obvious link between high GR expression and prostate cancer progression, as well as the frequent use of steroid hormones to mitigate symptoms in patients with advanced cancer, the potential effects of steroid hormones and their inhibitors on prostate cancer cells are of interest. Drugs being tested include Mifepristone, which exhibits partial AR agonism, but also more selective GR inhibitors such as Exicorilant and ORIC-101. So far, clinical trials evaluating these drugs in combination with enzalutamide for CRPC treatment have failed to yield significant clinical benefits [11, 59, 60]. However, these studies did not specifically select patients based on GR expression levels and it was proposed that only a subgroup of tumors with high GR levels, relying mainly on the GR-pathway for castration resistance, might respond to anti-GR treatment [11]. Considering the small cohorts of 39 and 41 patients in the selective GR inhibitor trials and a rate of only 11% strongly GR positive tumors as found in our study, this serves as a conclusive explanation for a poor overall treatment response.

It is of note that cancer morphology remains the strongest of all known predictors of prostate cancer prognosis. Already within traditional Gleason grade groups, a prognostic impact of GR expression was only found in Gleason 3 + 4 tumors. Based on the large cohort of prostate cancers available at our institution, we had earlier shown that Gleason grade information can be largely expanded by using the percentage of Gleason 4 patterns as a continuous variable. Both in biopsies and in prostatectomy samples, prostate cancer prognosis gradually deteriorates with increasing percentage of Gleason 4 patterns (quantitative Gleason grade) [61]. The lack of significant prognostic impact of GR staining intensity in any subgroups with a comparable quantitative Gleason grade demonstrates how difficult it is for biomarkers to outperform morphology-based malignancy grading in prostate cancer.

In summary, the results of our study demonstrate a pivotal role of GR expression in therapy-naïve prostate cancer. Given its strong and independent prognostic role, GR expression measurement may have clinical utility, most likely in combination with other markers. The potential role of GR as a predictive marker for the response to ADT and GR-targeting therapies remains to be further evaluated.

## Supplementary information

Supplementary Material

## Data Availability

All data generated or analyzed during this study are included in this published article.
