# Supplementary material for: High level expression of glucocorticoid receptor (GR) is linked to aggressive tumor features, early biochemical recurrence, and genetic instability in prostate cancer
Source: Prostate Cancer Prostatic Dis. 2025 Nov 5;29(1):181–8. doi: 10.1038/s41391-025-01046-8 (PMC12909130; doi:10.1038/s41391-025-01046-8)
Supplement: Supplementary file 1 — Supplementary Material [file 41391_2025_1046_MOESM1_ESM.pdf]

Supplementary Figure 1: Association between GR expression (negative, weak, moderate, strong) and biochemical recurrence in classical Gleason Grades, a) Gleason 3+3, b) Gleason 3+4, c) Gleason 4+3 and d) Gleason ≥4+4

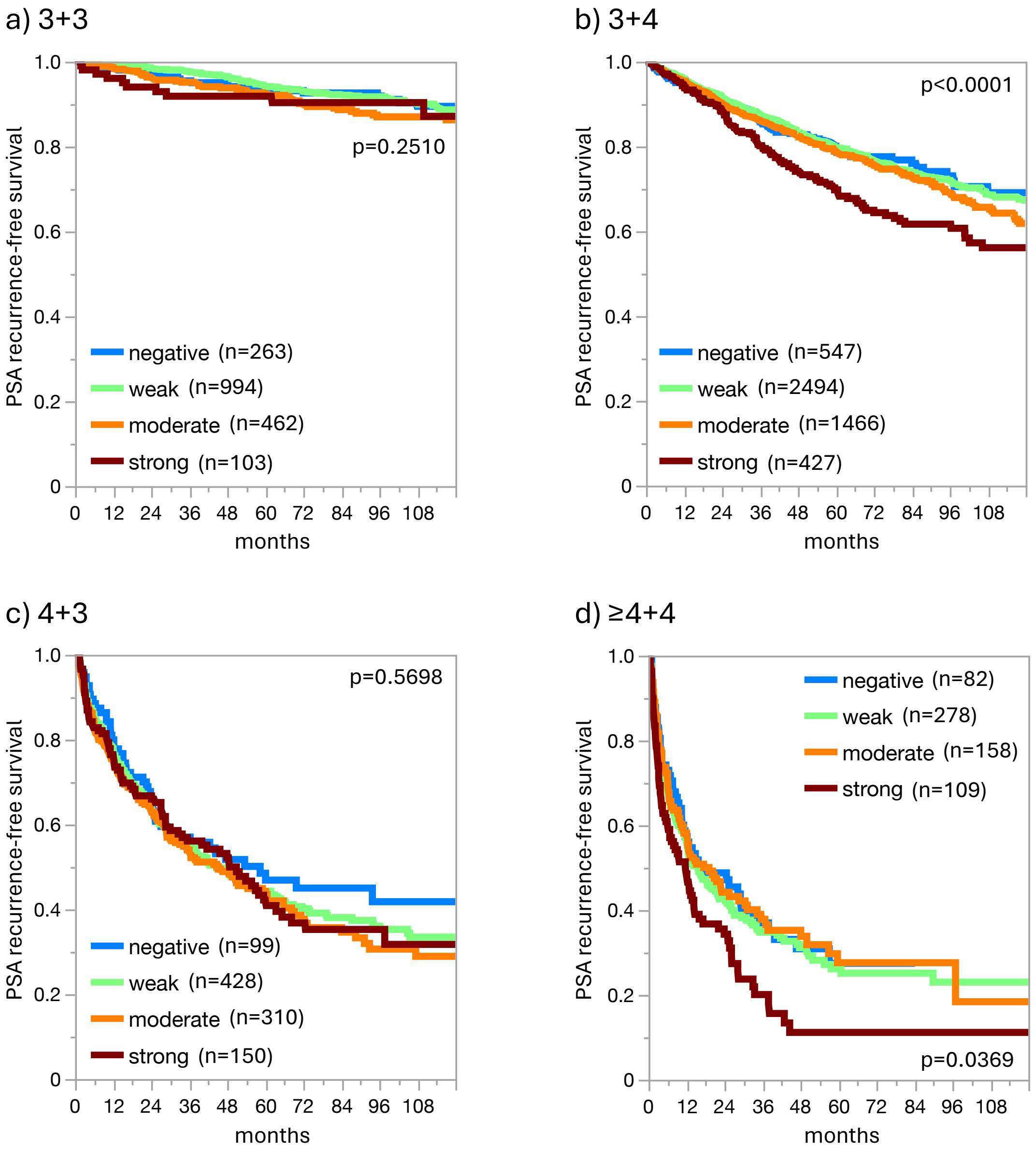

Supplementary Figure 2: Association between GR expression (negative, weak, moderate, strong) and biochemical recurrence in a) Gleason 3+3, b) Gleason 3+4 with <5% Gleason 4, c) 6-10% Gleason 4, d) 11-20% Gleason 4, e) 21-30% Gleason 4, f) 31-49% Gleason 4, g) Gleason 4+3 with 50-60% Gleason 4, h) 61-80% Gleason 4, i) >80% Gleason 4 and j) Gleason ≥4+4

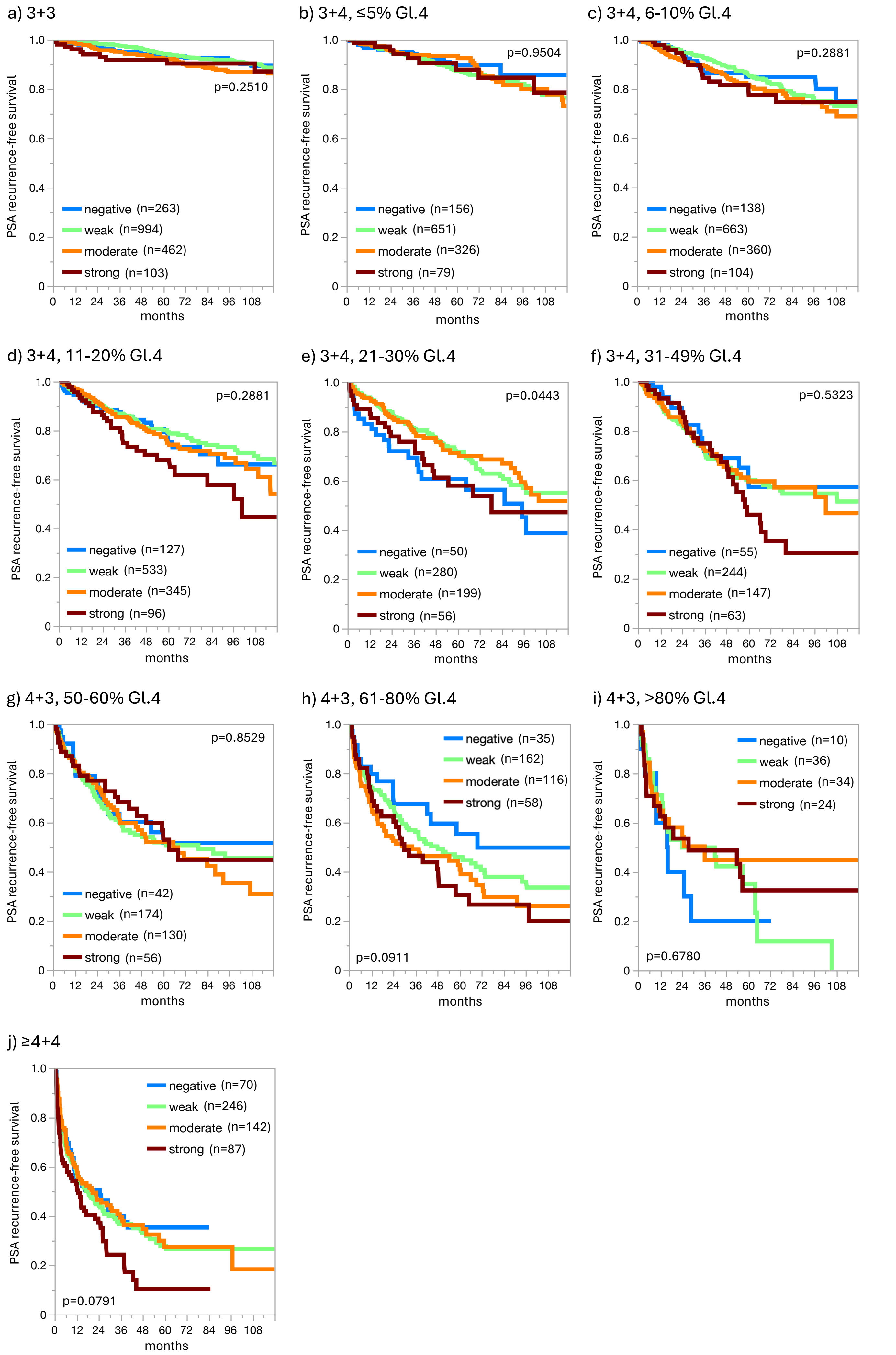

Supplementary Figure 3: Association between positive GR immunostaining and androgen receptor (AR) status in all cancer, ERG fusion negative and ERG fusion positive cancers

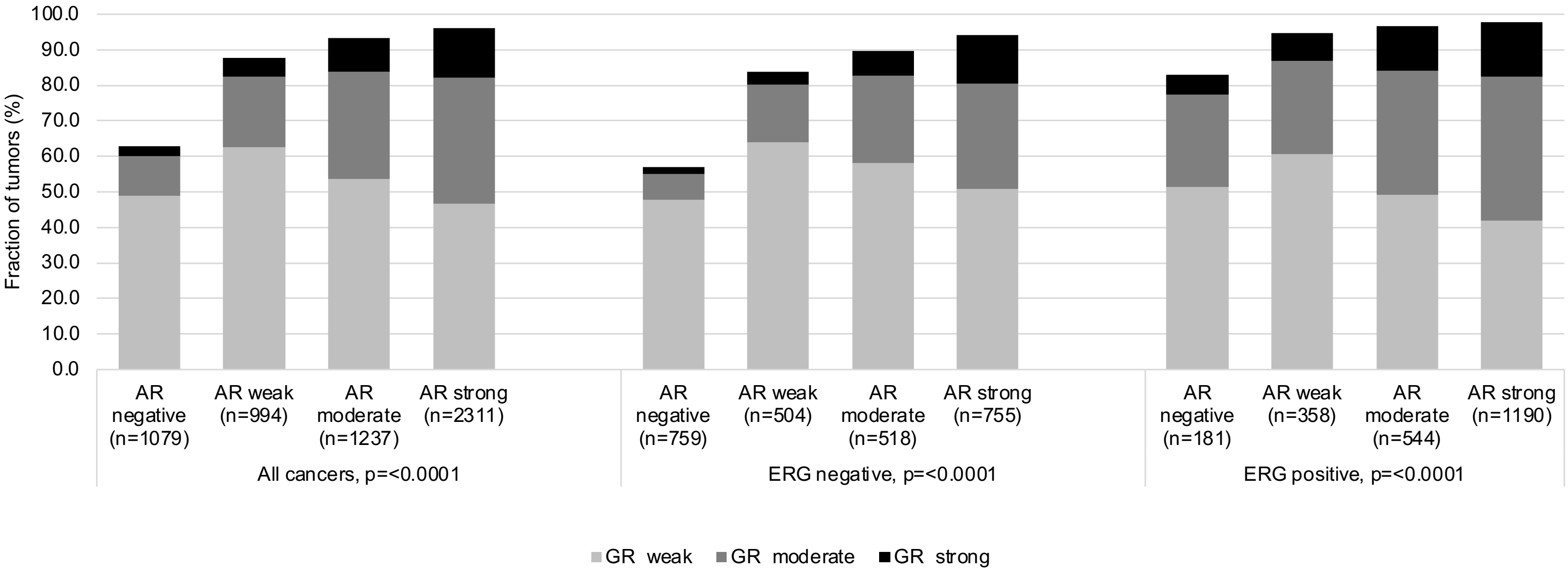

Supplementary Figure 4: Association between combined AR/GR expression and biochemical recurrence in all cancers

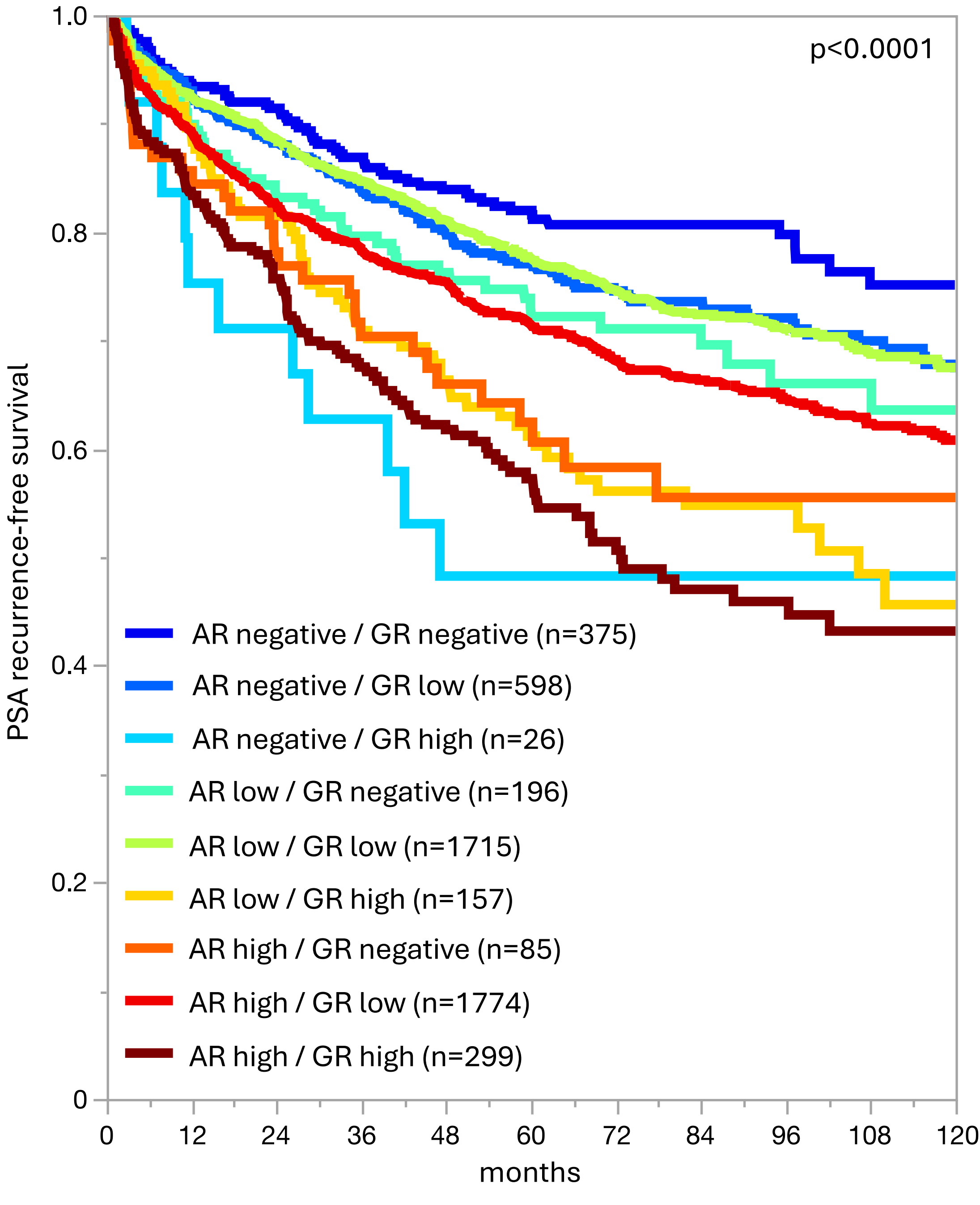

Supplementary Figure 5: Association between combined AR/GR expression and biochemical recurrence in cancers with a) negative, b) low and c) high AR expression

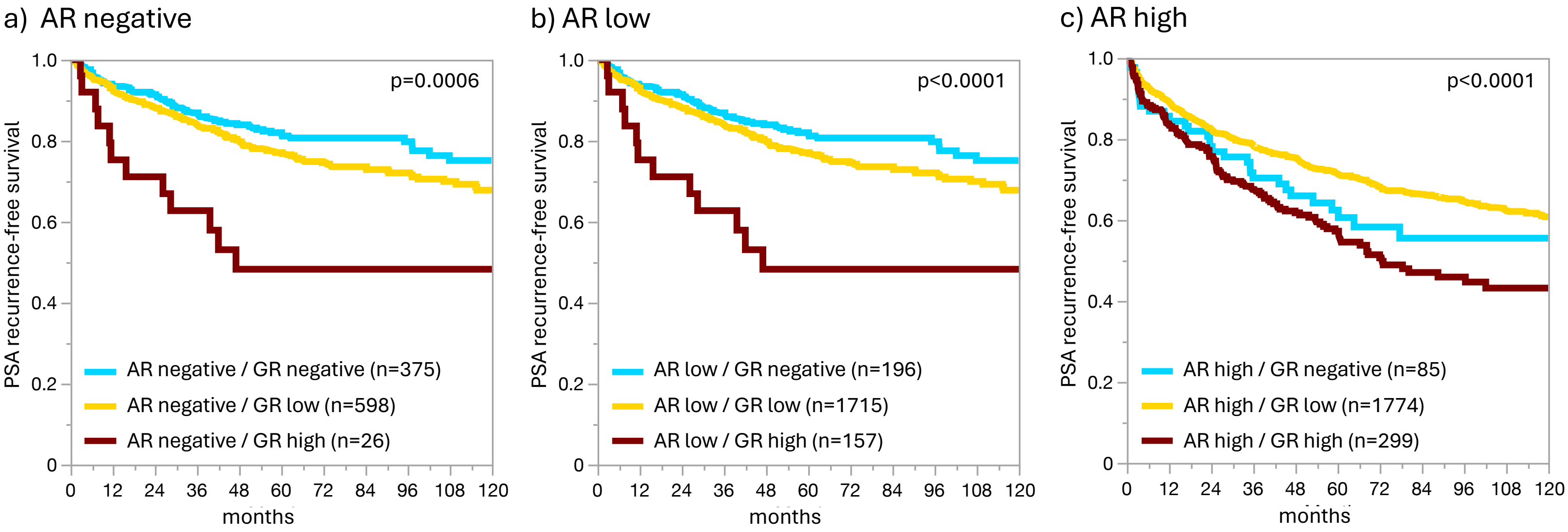

Supplementary Figure 6: Association between positive GR immunostaining and 3p13, 5q12, 6q15, 8p21, 10q23 (PTEN), 12p13, 16q24, 17p13 and 18q21 deletions in all, ERG negative and ERG positive cancers (correlated deletions only)

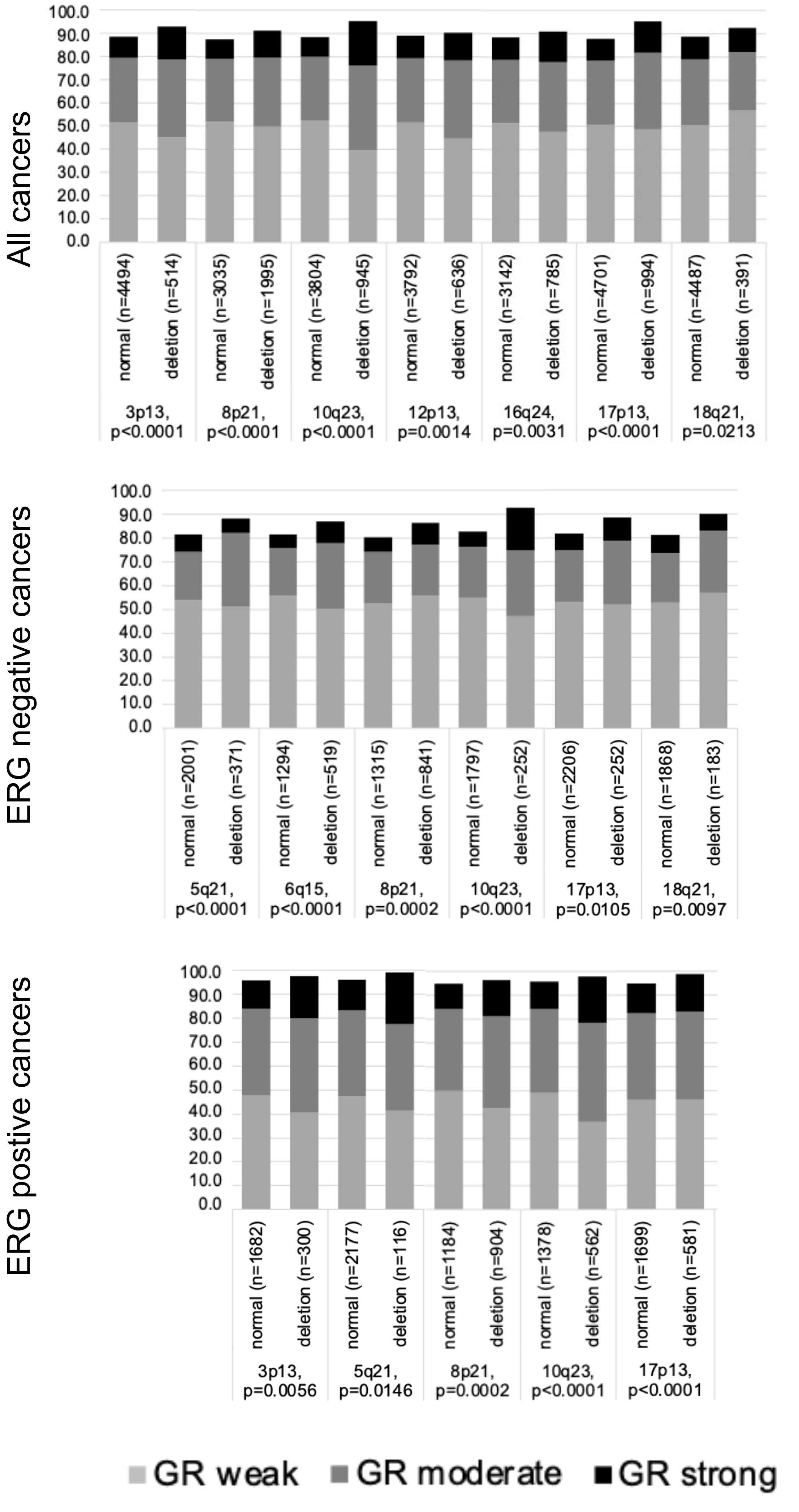

Supplementary Table 1: Pathological and clinical data of the arrayed prostate cancers

	**No. of patients (%)**	
	**Study cohort on TMA** (n=17,747)	**Biochemical relapse among categories**
**Follow-up (mo)**		
n	14464 (81.5%)	3612 (25%)
Mean	56.3	-
Median	48	-
Age (y)		
≤50	433 (2.4%)	66 (15.2%)
51-59	4341 (24.5%)	839 (19.3%)
60-69	9977 (56.4%)	2073 (20.8%)
≥70	2936 (16.6%)	634 (21.6%)
**Pretreatment PSA (ng/ml)**		
<4	2225 (12.6%)	313 (14.1%)
4-10	10520 (59.6%)	1696 (16.1%)
10-20	3662 (20.8%)	1043 (28.5%)
>20	1231 (7%)	545 (44.3%)
**pT stage (AJCC 2002)**		
pT2	11518 (65.2%)	1212 (10.5%)
pT3a	3842 (21.7%)	1121 (29.2%)
pT3b	2233 (12.6%)	1213 (54.3%)
pT4	85 (0.5%)	63 (74.1%)
**Gleason grade**		
≤3+3	3570 (18.1%)	264 (7.4%)
3+4	9336 (47.4%)	1436 (15.4%)
3+4 Tert.5	1697 (8.6%)	165 (9.7%)
4+3	2903 (14.7%)	683 (23.5%)
4+3 Tert.5	1187 (6%)	487 (41%)
≥4+4	999 (5.1%)	531 (53.2%)
**pN stage**		
pN0	10636 (89.4%)	2243 (21.1%)
pN+	1255 (10.6%)	700 (55.8%)
**Surgical margin**		
Negative	14297 (80.8%)	2307 (16.1%)
Positive	3388 (19.2%)	1304 (38.5%)

NOTE: Percent in the column "Study cohort on TMA" refers to the fraction of samples across each category. Percent in column "Biochemical relaps among categories" refers to the fraction of samples with biochemical relaps within each parameter in the different categories. Numbers do not always add up to 17,747 in the different categories because of cases with missing data. Abbreviation: AJCC, American Joint Committee on Cancer.

Supplementary Table 2: Association between GR immunostaining results and prostate cancer phenotype in ERG negative cancers

			GR IHC result				
		n evaluable	negative (%)	weak (%)	moderate (%)	strong (%)	p value
**all ERG negative cancers**		5359	16.8	51.7	23.5	8	
**Tumor stage**	pT2	3464	18.1	52.6	22.6	6.6	<0.0001
	pT3a	1127	15.6	50.9	24.7	8.8	
	pT3b-4	746	12.7	48	26.4	12.9	
**Gleason grade**	≤3+3	869	23	53.7	19.1	4.1	<0.0001
	3+4	2789	16.3	52.9	23.6	7.2	
	3+4 Tert.5	287	12.9	54	24	9.1	
	4+3	586	14	47.3	28	10.8	
	4+3 Tert.5	412	12.4	47.1	28.2	12.4	
	≥4+4	365	18.6	48.8	21.1	11.5	
**quantitative Gleason**	3+4 ≤5%	660	19.8	52.4	22.4	5.3	<0.0001
	3+4 6-10%	696	15.2	57.8	21	6	
	3+4 11-20%	618	17.2	51.9	23.6	7.3	
	3+4 21-30%	324	13.3	51.2	27.8	7.7	
	3+4 31-49%	278	15.1	54.3	20.5	10.1	
	4+3 50-60%	228	12.9	54	24	9.1	
	4+3 61-80%	208	14.9	49.1	24.6	11.4	
	4+3 >80%	68	12.5	46.6	29.3	11.5	
**Lymph node metastasis**	N0	3376	16.1	50.9	24.6	8.3	0.0031
	N+	401	13.5	46.4	26.4	13.7	
**Preop. PSA level (ng/ml)**	<4	518	11	56.2	20.8	12	<0.0001
	4-10	3032	16.3	51.3	24.4	8	
	11-20	1282	18.7	50.3	24.6	6.4	
	>20	499	21.4	52.5	18.8	7.2	
**Surgical margin**	negative	4218	16.8	52	23.7	7.5	0.0902
	positive	1124	17	50.2	23	9.8	

Supplementary Table 3: Association between GR immunostaining results and prostate cancer phenotype in ERG positive cancers

			GR IHC result				
		n evaluable	negative (%)	weak (%)	moderate (%)	strong (%)	p value
**all ERG positive cancers**		4407	3.8	44.5	37.1	14.5	
**Tumor stage**	pT2	2438	4.3	47.7	36.6	11.4	
	pT3a	1233	3.2	42.7	38.7	15.4	<0.0001
	pT3b-4	712	3.2	36.4	36.7	23.7	
**Gleason grade**	≤3+3	751	4.8	54.7	33.8	6.7	
	3+4	2403	4.1	45	38.6	12.3	
	3+4 Tert.5	188	3.2	42.6	37.2	17	<0.0001
	4+3	445	2.5	33.9	39.3	24.3	
	4+3 Tert.5	331	1.8	39.6	34.7	23.9	
	≥4+4	230	3.5	38.3	29.6	28.7	
**quantitative Gleason**	3+4 ≤5%	539	4.5	51	35.1	9.5	
	3+4 6-10%	604	4.1	47.7	36.9	11.3	
	3+4 11-20%	528	3.8	41.5	42.8	11.9	
	3+4 21-30%	287	4.2	42.9	40.1	12.9	<0.0001
	3+4 31-49%	228	4.4	40.8	37.7	17.1	
	4+3 50-60%	190	3.2	42.6	37.2	17	
	4+3 61-80%	142	2.1	32.1	43.7	22.1	
	4+3 >80%	32	4.9	32.4	39.4	23.2	
**Lymph node metastasis**	N0	2603	3.6	43.9	37.8	14.7	<0.0001
	N+	414	3.4	33.3	38.2	25.1	
**Preop. PSA level (ng/ml)**	<4	536	2.6	41.4	40.5	15.5	
	4-10	2627	3.7	46.3	36.7	13.4	0.0315
	11-20	888	4.5	41.9	36.8	16.8	
	>20	318	5.3	41.8	36.8	16	
**Surgical margin**	negative	3358	4	44.6	37.8	13.5	<0.0001
	positive	1026	3.3	43.8	35.1	17.8	

Supplementary Table 4: Multivariate analysis including GR expression in all cancers, ERG negative and ERG positive cancers

Tumor subset	Scenario	n analyzable	p -value							
			preoperative PSA-Level	pT Stage	cT Stage	Gleason grade prostatectomy	Gleason grade biopsy	pN stage	R status	GR-Expression
all cancers	1	6428	<.0001*	<.0001*	-	<.0001*	-	<.0001*	<.0001*	0.0015
	2	9503	<.0001*	<.0001*	-	<.0001*	-	-	<.0001*	<0.0001
	3	9350	<.0001*	-	<.0001*	<.0001*	-	-	-	<0.0001
	4	7903	<.0001*	-	<.0001*	-	<.0001*	-	-	<0.0001
ERG negative cancers	1	2831	<.0001*	<.0001*	-	<.0001*	-	<0.0001*	0.4837	0.0179
	2	4170	<.0001*	<.0001*	-	<.0001*	-	-	0.0071	0.0006
	3	4112	<.0001*	-	<.0001*	<.0001*	-	-	-	<0.0001
	4	3428	<.0001*	-	<.0001*	-	<0.0001	-	-	0.0003
ERG postive cancers	1	2316	<.0001*	<.0001*	-	<.0001*	-	0.0029	0.0002	0.1382
	2	3498	<.0001*	<.0001*	-	<.0001*	-	-	<.0001*	0.1496
	3	3428	<.0001*	-	<.0001*	<.0001*	-	-	-	0.067
	4	2931	<.0001*	-	<.0001*	-	<.0001*	-	-	0.0006
